# Cessation Outcomes Among Quitline Callers in Three States During a National Tobacco Education Campaign

**DOI:** 10.5888/pcd12.150024

**Published:** 2015-07-16

**Authors:** Katrina A. Vickerman, Lei Zhang, Ann Malarcher, Paul Mowery, Chelsea Nash

**Affiliations:** Author Affiliations: Lei Zhang, Ann Malarcher, Office on Smoking and Health, Centers for Disease Control and Prevention, Atlanta, Georgia; Paul Mowery, Biostatistics, Inc, Atlanta, Georgia; Chelsea Nash, Alere Wellbeing, Seattle, Washington.

## Abstract

**Introduction:**

Antismoking mass media campaigns, such as the Centers for Disease Control and Prevention’s Tips from Former Smokers (Tips) campaign, increase the number of tobacco users calling tobacco quitlines. Few studies have investigated long-term tobacco use cessation for callers during antismoking media campaigns. Studies have suggested that callers during campaigns may be less committed to quitting and have lower quit rates. This study examines tobacco user cessation outcomes 7 months after quitline enrollment during the 2012 Tips campaign (March 19 through June 10, 2012).

**Methods:**

We analyzed data for 715 tobacco users who enrolled in the Nebraska, North Carolina, or Texas state quitline multiple-call programs during the 2012 Tips campaign and responded to a 7-month postenrollment survey (38.5% survey response rate). We used multivariable logistic regression analyses to determine whether 7-day and 30-day point prevalence abstinence rates 7 months after enrollment were related to level of exposure to the campaign.

**Results:**

In multivariable models, only lower nicotine dependence and higher call completion were associated with higher odds of 7-day and 30-day abstinence 7 months after enrollment. Tips campaign exposure was not associated with abstinence.

**Conclusion:**

Once enrolled in quitline counseling, quitline callers achieved similar outcomes regardless of Tips campaign exposure levels. While the campaign did not appear to directly affect odds of tobacco abstinence through quitlines, antismoking mass media campaigns such as Tips are valuable in increasing tobacco users’ exposure to quitlines and thus increasing their likelihood of making a quit attempt and eventually achieving tobacco abstinence.

## Introduction

Smoking continues to burden society with over 480,000 premature deaths, $156 billion in lost productivity, and $133 billion in direct medical care expenditures in the United States annually (1). To educate the public about health consequences of tobacco use, encourage cessation, and provide information on free resources to aid in quitting, the Centers for Disease Control and Prevention (CDC) launched the first Tips from Former Smokers (Tips) national media campaign on March 19, 2012. Approximately two-thirds of Tips advertisements were tagged with the national quitline routing number 1-800-QUIT-NOW. Quitlines are cost-effective behavior change programs that can increase the odds of tobacco use cessation by 60% compared with unassisted attempts (2,3).

The Tips campaign successfully increased calls to 1-800-QUIT-NOW, visits to smokefree.gov, and population-level quit attempts (4,5). Among 23 state quitlines, call volumes increased 89%, quit attempts reported during treatment increased 25%, and 7-day quits reported during treatment increased 22%, although proportionally fewer callers made an attempt or quit for 7 days compared with the same time period in 2011 (6). These findings align with research from other antismoking mass media campaigns that found that evidence-based advertisements increased call volumes to quitlines and, in some studies, increased short-term quitting behaviors such as quit attempts (7–11). However, studies on longer-term quit outcomes are limited. Findings from the 2007–2008 EX Campaign revealed that quit rates at 6-month follow-up did not significantly differ based on campaign awareness (10). In a Massachusetts study, higher potential exposure to antismoking advertisements in the 24 months before a baseline assessment increased the odds of a smoker being abstinent approximately 2 years later at follow-up; exposure to emotionally evocative advertisements appeared to drive this effect, whereas a Wisconsin study found that reported exposure to “keep-trying-to-quit” and secondhand smoke advertisements was not related to abstinence 1 year later (12,13). Given these mixed results, more research is needed to understand how antismoking messages relate to long-term quit outcomes and the role of quitline cessation treatments in this relationship.

The objective of our study was to determine whether quit outcomes 7 months after enrollment were related to Tips campaign exposure among tobacco users seeking help with quitting from state quitlines. We hypothesized that the Tips campaign may have influenced callers and their quit outcomes in 2 possible directions. First, greater exposure to Tips messaging may have increased the likelihood that quitline enrollees successfully quit. Alternatively, greater exposure may have prompted callers who were less ready or less motivated to quit to engage in quitline treatment; these callers may have had greater difficulty quitting and remaining quit (14).

## Methods

In this observational study, we examined whether estimated levels of Tips exposure, as measured by television gross rating points (GRPs), were related to 7-day and 30-day quit outcomes assessed at 7-month evaluations among quitline callers in 3 states: Nebraska, North Carolina, and Texas. These quitlines were chosen because they provided multiple-call programs, the states agreed to participate in the study, and the quitlines are operated by, and had evaluations conducted by, the same quitline vendor, Alere Wellbeing, during the Tips campaign from March 19 through June 10, 2012. The Western Institutional Review Board reviewed this study and determined that it met requirements for waiver of consent. 

### The Tips campaign

The Tips campaign is the first federally funded, national tobacco education campaign. Tips’ evidence-based, emotionally evocative ads featured former smokers talking about their experiences and their families’ experiences living with diseases caused by smoking and secondhand smoke exposure (15). The campaign ran for 12 weeks with ads on television in all US media markets via a national purchase of commercial advertisement time on cable networks. Additionally, 49 media markets were selected to receive a heavy-up local buy (ie, high concentration of advertising in a local area). Tips advertising was conducted predominately via television, but also via radio, online video and banners, print media, and out-of-home advertising. To provide resources for quitting, advertisements were tagged with either the toll-free, national quitline number, which connects callers directly to their state tobacco quitline, or the National Cancer Institute’s smokefree.gov website, which promoted 1-800-QUIT-NOW. Telephone-based tobacco quitlines are available in all 50 states and the District of Columbia.

### Quitline programs in Nebraska, North Carolina, and Texas

Nebraska, North Carolina, and Texas all offered a multiple-call telephone-based cessation program during the study timeframe. The multiple-call program included an initial assessment and planning call plus 3 to 4 additional outbound counseling calls and was based on the US Public Health Service clinical practice guidelines (2) and social cognitive theory (16). All Nebraska and North Carolina callers who said they were ready to quit in the next 30 days were eligible for the multiple-call program. Texas offered the multiple-call program to all uninsured and Medicaid-insured callers and to all tobacco users in their designated Tobacco Prevention and Control Coalition (TPCC) counties (callers with private insurance in non-TPCC counties were only eligible for a Web-based cessation program). Additional details regarding quitline services provided in Nebraska, North Carolina, and Texas during the study period, including nicotine replacement therapy (NRT) benefits offered through the quitlines, are presented in Table 1. Other service and data collection procedures were the same for the 3 state quitlines.

**Table 1:** Quitline Services for Tobacco Users Enrolled in the Nebraska, North Carolina, or Texas State Tobacco Quitlines, March 19–June 10, 2012

Service	Nebraska	North Carolina	Texas
**1-call program**	All tobacco users	All tobacco users	Not offered
**Multiple-call program^a^**	5-Call program for tobacco users ready to quit in the next 30 days	4-Call program for tobacco users ready to quit in the next 30 days	5-Call program for the following groups: • Uninsured • Medicaid-insured • Callers in designated Tobacco Prevention and Control Coalition (TPCC) counties (awarded enhanced services via a state-based competitive request-for-proposal process)
**10-Call program**	Pregnant tobacco users	Pregnant tobacco users	Pregnant tobacco users
**Web Coach** b	All telephone program participants	All telephone program participants	All telephone program participants
**Stand-alone Web-based tobacco cessation program**	Not offered	For tobacco users who preferred to receive only online support	For tobacco users who preferred to receive only online support Only service option for tobacco users not eligible for telephone program (commercially insured in non-TPCC counties)
**Direct mail order NRT**	Not offered Proof of quitline enrollment and completion of a program call was a component for some Medicaid participants to receive NRT or medications through their pharmacy benefits manager	8-Week supply of patches, lozenges, or gum for multiple- call program enrollees (through May 20, 2012) 8-Week supply of patches for multiple- call program enrollees with state employees’ health insurance (duration of study timeframe)	8-Week supply of patches, gum, or lozenges for MC enrollees who were uninsured or pregnant Benefit extended to all enrollees who were fax-referred to the quitline from medical clinics starting March 30, 2012

Abbreviation: NRT, nicotine replacement therapy.

a Initial assessment and planning call plus 3 to 4 outbound calls.

b An interactive online tool to complement telephone coaching (29).

### Sample selection

Tobacco users (ie, cigarette, cigar, and pipe smokers and users of smokeless tobacco and other tobacco products) who were enrolled in their state’s quitline telephone program were randomly selected for the Texas and Nebraska evaluations. For the North Carolina evaluation, Medicaid-insured participants were oversampled by using a probability sampling scheme. Participants in all evaluations were English or Spanish speaking (survey was conducted in participant’s preferred language), aged 18 years or older, consented to evaluation follow-up during program registration, were enrolled in the 1-call or multiple-call cessation program, and completed a coaching call.

Trained interviewers conducted follow-up surveys by telephone. One or more attempts were made to reach a participant each day on up to 11 attempt days over approximately 4 weeks. The North Carolina evaluation also used Web-based surveying. For the North Carolina evaluation, participants who provided an email address and consented to be contacted by email were first emailed a link to the 7-month survey and invited to participate. Participants who did not complete the Web-based survey after 3 reminders were then contacted for the telephone-based survey.

This study included only multiple-call program enrollees because only 11 one-call enrollees were selected for and completed the follow-up survey. Across the 3 states, 1,908 tobacco users who enrolled in the multiple-call programs from March 19 through June 10, 2012, were selected for evaluation; 734 completed the 7-month survey for a response rate of 38.5%. Our final sample was 715 participants who provided a valid answer (ie, other than refused to answer or responded “don’t know”) to the survey question, “When did you last use tobacco, even a puff or a pinch? (Please do not include electronic cigarettes.)”

### Measures

Demographics (age, sex, race/ethnicity, education, and health insurance status), time to first use of tobacco after waking, and cigarettes smoked per day were collected during program registration. We created an index to represent nicotine dependence based on cigarettes smoked per day and time to first use. Time to first use was reported on a 4-point scale: 1) 61 or more minutes, 2) 31 to 60 minutes, 3) 6 to 30 minutes, and 4) within 5 minutes. Cigarettes smoked per day were reported on a continuous scale and categorized into 4 groups: 1) 0 to 10 cigarettes, 2) 11 to 20 cigarettes, 3) 21 to 30 cigarettes, and 4) 31 or more cigarettes (17). The mean of the two 4-point scales was used to create the index.

During the 7-month survey, participants reported when they had last used tobacco and whether they had used any medications to help them quit since registering for the program (ie, nicotine patches, gum, lozenges, spray, or inhaler; varenicline; bupropion; or other medications not FDA-approved for tobacco cessation). The 7-day and 30-day point prevalence tobacco abstinence rates were calculated from participants’ reports of when they last used tobacco.

Exposure to the Tips campaign was measured by Tips television GRPs in each caller’s designated market area. Television GRPs are defined as the product of media reach (ie, the percentage of audience that is exposed to a given advertisement) and frequency (ie, the number of times the audience is exposed to an advertisement) during a given period of time (15). Television GRP exposure was computed by summing the weekly GRPs in the caller’s designated market area (on the basis of the area code of the caller’s telephone number) during the 12 weeks of the campaign. In this sample, callers’ average estimated exposure to the Tips campaign was 1,781 television GRPs (SD, 647.6; median, 1,861; range, 802–3,403).

### Analyses

All analyses were conducted in SAS, version 9.3 (SAS Institute Inc). To reduce survey response bias, data were weighted for nonresponse based on age, sex, race/ethnicity, and insurance. Poststratification weights were used to adjust for the Medicaid oversample in North Carolina’s evaluation to appropriately reflect the overall sample eligible for the evaluation. Weights were computed using a raking macro (18).

Participant characteristics, call completion, and medication use data were presented for callers below and above the sample median television GRP exposure level. *P* values were computed for categorical and continuous variables by using SAS’s Surveyreg and Surveylogistic procedures, accounting for clustering by state. Differences in quit outcomes for callers below and above the median television GRP exposure level were examined using SAS’s Proc Surveylogistic, accounting for clustering by state. Multivariable logistic regression analyses examining continuous television GRP exposure (predictor) and 7-day and 30-day point prevalence abstinence rates (outcomes) were conducted with caller demographics, baseline dependence level, call completion, and reported use of cessation medications during the program included as control variables and the state included as a fixed effect to account for differences in services and tobacco control environment by state.

We conducted 2 alternative analyses to examine the impact of 1) including education in the model, which was not used in the primary model because it appeared that missing data on this variable (5% of sample) may not have been at random, and 2) including a quadratic term for television GRPs to test for nonlinear effects of campaign exposure on quit status. Results from these alternative models were the same as results for the primary model.

## Results

Quitline callers with higher Tips exposure were more likely to be female and non-Hispanic white (Table 2). No significant differences were identified in age, education, insurance type, nicotine dependence, call completion, or use of cessation medications reported at follow-up. At the time of the 7-month survey, 27.7% of participants reported tobacco abstinence for 7 or more days and 24.5% reported abstinence for 30 or more days. These quit rates did not differ between callers with below-median versus above-median estimated Tips exposure in bivariate analyses.

**Table 2:** Characteristics of Callers (N = 715) and Quit Rates Evaluated at 7-Month Follow-Up by Tips Television Gross Rating Points^a^ Exposure Among Tobacco Users Enrolled in the Nebraska, North Carolina, or Texas State Tobacco Quitline Multiple-Call Programs, March 19–June 10, 2012

Characteristic	Total (N = 715)	Television Gross Rating Points^a^ Below Median (802–1,835; n = 351)	Television Gross Rating Points^a^ Above Median (1,887–3,403; n = 364)	*P* Value^b^
**Sex, female, %**	64.9	63.3	66.4	.04
**Age, y, mean (SD)**	44.2 (12.9)	42.4 (13.5)	45.9 (12.2)	.09
**Education, %**				
Less than high school graduate	23.4	25.6	21.2	.73
GED/high school graduate	31.5	30.9	32.0	
More than high school graduate	45.2	43.4	46.8	
**Race/ethnicity, %**				
Non-Hispanic white	63.6	56.6	70.4	<.001
Hispanic or other	36.4	43.4	29.6	
**Insurance type, %**				
Uninsured	42.8	51.4	34.4	.51
Medicaid	33.9	30.7	37.0	
Private insurance (including Medicare)	23.3	17.9	28.7	
**Nicotine dependence index at registration** c **, %**				
Low dependence (0–2.5)	55.3	56.0	54.6	.73
High dependence (3,4)	44.7	44.0	45.4	
**Counseling calls completed, mean (SD)**	2.1 (1.4)	2.1 (1.5)	2.1 (1.4)	.996
**Used quit medication since enrollment (reported on 7-month survey), %**	75.5	74.7	76.3	.13
**Self-reported point-prevalence quit rates at 7-month follow-up, %**				
% Quit 7 days (95% CI)	27.7 (23.7–32.1)	26.9 (22.0–32.3)	28.6 (23.6–34.1)	.64
% Quit 30 days (95% CI)	24.5 (20.8–28.6)	24.0 (19.2–29.6)	24.9 (20.2–30.4)	.80

Abbreviations: CI, confidence interval; GED, general education development; SD, standard deviation; Tips, Tips from Former Smokers campaign.

a Television gross rating points are the product of media reach (ie, the percentage of audience that is exposed to a given advertisement) and frequency (ie, the number of times the audience is exposed to an advertisement) during a given period of time (15). The sample median television gross rating points exposure of 1,861 was used to define the below-median and above-median groups.

b All *P* values were adjusted for state clustering. Logistic regression was used to compute *P v*alues and confidence intervals for categorical variables. Regression was used to compute *P* values for continuous variables.

c A 4-point scale index to represent nicotine dependence level based on cigarettes per day and time to first tobacco use after waking. Higher scores on the index represent a higher level of nicotine dependence.

In multivariable logistic regression models, only nicotine dependence level and number of counseling sessions completed were significantly associated with 7-day and 30-day abstinence rates at the 7-month evaluation. Callers with high dependence scores were less likely to be quit (quit ≥7 days model, adjusted OR, 0.71 [95% CI, 0.57–0.89], *P* = .003; quit ≥30 days model, adjusted OR, 0.74 [95% CI = 0.58–0.93], *P* = .01) than those with lower scores, and callers who completed more counseling calls were more likely to be quit (quit ≥7 days model, adjusted OR, 1.15 [95% CI, 1.01–1.30), *P* = .03; quit ≥30 days model, adjusted OR, 1.15 (95% CI, 1.01–1.30), *P* = .03) than those who completed fewer calls. Tips television GRPs were not associated with abstinence at follow-up (Table 3).

**Table 3:** Multivariable Models of the Relationship of Tips Television Gross Rating Points^a^ Exposure Group and 7-Day and 30-Day Abstinence Among Tobacco Users Enrolled in the Nebraska, North Carolina, or Texas State Tobacco Quitline Multiple-Call Programs, March 19, 2012–June 10, 2012

Characteristic	Quit ≥7 Days (N = 634)		Quit ≥30 Days (N = 634)	
	Adjusted OR (95% CI)	*P* Value^a^	Adjusted OR (95% CI)	*P* Value^a^
**Age**	1.00 (0.99–1.02)	.64	1.00 (0.99–1.02)	.99
**Sex**				
Male	1 [Reference]	.37	1 [Reference]	.62
Female	0.84 (0.58–1.23)		0.91 (0.61–1.34)	
**Insurance status**				
Private insurance (including Medicare)	1 [Reference]	.09	1 [Reference]	.07
Medicaid	0.68 (0.42–1.11)		0.62 (0.38–1.03)	
Uninsured	0.59 (0.37–0.95)		0.58 (0.35–0.94)	
**Race/ethnicity**				
Non-Hispanic white	1 [Reference]	.60	1 [Reference]	.92
Hispanic or other	0.90 (0.61–1.34)		0.98 (0.65–1.47)	
**Nicotine dependence index^b^**	0.71 (0.57–0.89)	.003	0.74 (0.58–0.93)	.01
**Use of cessation medications**				
No	1 [Reference]	.40	1 [Reference]	.27
Yes	1.21 (0.78–1.87)		1.30 (0.82–2.05)	
**Number of counseling sessions completed**	1.15 (1.01–1.30)	.03	1.15 (1.01–1.30)	.03
**Television gross rating points^c^ exposure**	0.99 (0.95–1.02)	.40	0.99 (0.96–1.03)	.59

Abbreviations: CI, confidence interval; OR, odds ratio; Tips, Tips from Former Smokers campaign.

a Multivariable logistic regression was used to compute *P* values. Model also controlled for state as a fixed effect. Callers with missing data on 1 or more model variables were excluded from the model.

b A 4-point scale index to represent nicotine dependence level based on cigarettes per day and time to first tobacco use after waking. Higher scores on the index represent a higher level of nicotine dependence.

c Television gross rating points are the product of media reach (ie, the percentage of audience that is exposed to a given advertisement) and frequency (ie, the number of times the audience is exposed to an advertisement) during a given period of time (15). Tips television gross rating points were entered as a continuous variable and rescaled so that the odds ratios reflect change with each 100-point increase in television gross rating points.

## Discussion

We examined whether 7-month quit outcomes were related to Tips campaign exposure among tobacco users seeking help with quitting from state quitlines. Quitting at 7 months following quitline enrollment did not differ by the caller’s estimated exposure to the 2012 Tips campaign on the basis of their designated media market in the 3 states studied. In multivariable models examining demographic characteristics, baseline nicotine dependence, quitline program use, and television GRP exposure, only a higher number of program calls completed and a lower baseline nicotine dependence were significant predictors of quit status at follow-up, which aligns with previous research identifying dependence and engagement as important predictors of abstinence (19–22).

Few previous studies have examined longer-term quit outcomes in relation to media campaign exposure. Population-level studies have had mixed results with regard to ad exposure being linked to cessation, with 1 study showing positive results for emotionally evocative ads and 2 studies finding no relationship between cessation and how-to-quit, keep-trying-to-quit, or secondhand smoke ads (10,12,13). Our study differed from this previous work in that we examined cessation for callers who enrolled in telephone-based cessation services through state quitlines during a national tobacco education campaign promoting the national quitline portal as a quitting resource on many of the ads. For quitlines to have a measurable impact on cessation rates at the population level, they must be able to reach a sufficient proportion of the tobacco user population in addition to engaging callers in effective interventions (ie, impact = effectiveness × reach) (23). The Tips campaign was effective in driving increased numbers of callers (reach) to treatment in previous studies; this study provided evidence that callers seeking treatment during the Tips campaign had similar long-term cessation outcomes (effectiveness) (5,6,24). In other words, once enrolled in quitline treatment, program participation and tobacco use characteristics such as baseline nicotine dependence are predictors of success, whereas level of campaign exposure was not a significant predictor. Together with the previous research, our findings indicate that antismoking mass media campaigns such as Tips can promote and facilitate quitting with assistance from quitlines by increasing quitline exposure.

We reported previously on intermediate cessation outcomes during treatment in a sample of 23 state quitlines for Tips callers (6). When examining quit attempts and 7-day quits reported on treatment calls among multiple-call program enrollees who completed at least 1 counseling call, the relationship between Tips television GRPs and intermediate cessation outcomes varied by per capita tobacco control program (TCP) funding at the state level (6). In high TCP funding states, high television GRP exposure (≥2,000) was related to a greater likelihood of making a quit attempt and reporting a 7-day or longer quit. However, in low TCP funding states, the highest exposure group (≥2,000 television GRPs) was less likely to report being quit for 7 or more days during treatment calls compared with the middle exposure group (1,200–1,999 television GRPs). We hypothesized that previous tobacco control efforts in high TCP funding states may have prepared callers in those states to be more ready to quit, whereas callers in low TCP funding states may have had a less supportive quitting environment or been less advanced on the readiness-to-quit continuum. Given that our 3 study states were all at or below the median in terms of their per capita tobacco control funding, it is encouraging that callers in higher Tips exposure markets (compared with callers in lower exposure markets) were not less likely to be quit at 7-month follow-up even though they might be less ready to quit (14,25,26). Additionally, the 2012 Tips campaign was designed to deliver increased television GRPs to some high smoking prevalence areas, which may have biased our results toward nonsignificant findings given that these high-prevalence areas may have made less progress in tobacco control strategies that would create a supportive environment for a caller’s quitting process, such as smoke-free policies and antitobacco norms. For this reason, it is also reassuring that callers in designated market areas with higher Tips exposure were not less likely to be quit at 7-month follow-up than those in lower Tips exposure market areas.

Several study limitations should be noted. First, adequate data were not available for another time period (eg, the previous year) to serve as a comparison to the quit rates observed during the Tips campaign. Additionally, callers during the months just before or after the campaign could not provide a valid comparison because these callers may have been exposed to the campaign before reporting on 7-month cessation outcomes. Thus, there is not a no-exposure condition in this study, because all designated market areas had substantial levels of ad exposure. However, the quit outcomes for these states (30-day point prevalence abstinence rate of 24.5%) during the Tips campaign are in line with 7-month outcomes reported for other state quitline samples (22,27). Second, although a standard method of examining campaign exposure, television GRPs reflect estimated population-level exposure to a media campaign and do not necessarily reflect an individual’s exposure to the campaign. Third, our response rate was low (38.5%), but typical of studies following up with quitline callers (22,28). Fourth, 7-month evaluations for callers during the Tips campaign were available for only 3 states, which limited the generalizability of our study. Findings from these states may be particularly relevant to other states at or below the median per capita tobacco control funding level.

Despite these limitations, our study represents the first to examine the impact of a national tobacco education campaign on long-term cessation outcomes among tobacco users seeking cessation treatment. Together with previous research, these findings suggest that despite the possibility that tobacco users seeking treatment from quitlines during the Tips campaign might be less committed to quitting, callers achieved similar outcomes regardless of campaign exposure levels once they were enrolled in quitline counseling (14,25). While the campaign did not appear to directly affect odds of tobacco abstinence through quitlines, antismoking mass media campaigns such as Tips are valuable in increasing tobacco users’ exposure to quitlines and thus increasing their likelihood of making a quit attempt and eventually achieving tobacco abstinence.
